# Quantifying Dependence Between Random Vectors: A New Index with Applications

**DOI:** 10.3390/e28101105

**Published:** 2026-10-10

**Authors:** Xiaoqian Zhang, Chuancun Yin

**Affiliations:** School of Statistics and Data Science, Qufu Normal University, Qufu 273165, China; xiaoqianzhang@qfnu.edu.cn

**Keywords:** characteristic function, distance covariance, independence, limit theorems, sub-independence, 62G10, 62H20, 62G20, 60E10

## Abstract

This article proposes a new index for quantifying the degree of dependence between random vectors with the same dimension. The index takes values in [0, 1] and equals zero if and only if the random vectors are sub-independent. Unlike mere uncorrelatedness, sub-independence implies a stronger form of dependence while remaining strictly weaker than full independence. The proposed index is constructed via characteristic functions and admits a simplified representation in terms of moments. We establish its theoretical properties and derive a computationally efficient formula for the corresponding empirical measure. Furthermore, we investigate the asymptotic behavior and finite-sample performance of the estimator and demonstrate its practical utility through applications in machine learning, actuarial science, and renewal theory. A numerical comparison with Pearson correlation and squared distance correlation is also given under several dependence structures. We also give a real-data application to international stock and foreign exchange returns.

## 1. Introduction

In the era of big data, measuring and testing the dependence between random vectors plays a central role in many fields, including statistics, biology and engineering, data science, and machine learning, among others. To date, several methods for measuring and testing dependence have appeared in the literature. The most popular correlation coefficient is the Pearson product–moment correlation, which measures the linear dependence between two random variables. The other two most popular classical measures of statistical association are Spearman’s ρ and Kendall’s τ. These coefficients are very powerful for detecting linear or monotone associations; however, they do not fully characterize dependence. To overcome those limitations, Székely, Rizzo, and Bakirov [1] proposed a nonparametric distance covariance (dCov) and distance correlation (dCor) to measure dependence between two random vectors of arbitrary dimensions, not necessarily the same dimension; see also Székely and Rizzo [2]. Moreover, they also extended it to a more general class of measures called α-distance dependence measures. Further, Székely and Rizzo [3] proposed a *t*-test based on a modified distance covariance. The research progress in this topic can be found in recent papers by Shao and Zhang [4], Yao et al. [5], Zhu et al. [6], Li et al. [7], Borgonovo et al. [8], Liu and Shang [9], and the references therein. For two random vectors $X\in R^{p}$ and $Y\in R^{q}$, according to Székely, Rizzo, and Bakirov [1], the α-distance (0<α<2) covariance between *X* and *Y* is defined as

(1) $$\begin{array}{c} {\mathrm{dCov}}^{2(\alpha )}\left(X,Y\right)=\frac{1}{c(p,\alpha )c(q,\alpha )}\int_{R^{p+q}}\frac{|\phi_{X,Y}\left(t,s\right)-\phi_{X}\left(t\right)\phi_{Y}{\left(s\right)|}^{2}}{{\left|t\right|}_{p}^{\alpha +p}{\left|s\right|}_{q}^{\alpha +q}}dtds, \end{array}$$

where $\phi_{X}\left(t\right)$ and $\phi_{Y}\left(s\right)$ are the characteristic functions of *X* and *Y*, respectively, $\phi_{X,Y}$ is the joint characteristic function of (X,Y), and

$$c\left(p,\alpha \right)=\frac{2\pi^{p/2}\Gamma \left(1-\alpha /2\right)}{\alpha 2^{\alpha }\Gamma \left(\left(\alpha +p\right)/2\right)},\;\;c\left(q,\alpha \right)=\frac{2\pi^{q/2}\Gamma \left(1-\alpha /2\right)}{\alpha 2^{\alpha }\Gamma \left(\left(\alpha +q\right)/2\right)}.$$

Here, Γ(·) is the complete gamma function. In the simplest case, α=1, the constants in (1) become

$$c_{p}=c\left(p,1\right)=\frac{\pi^{(1+p)/2}}{\Gamma ((1+p)/2)},\;\;c_{q}=c\left(q,1\right)=\frac{\pi^{(1+q)/2}}{\Gamma ((1+q)/2)},$$

which are half the surface areas of the unit spheres in $R^{p}$ and $R^{q}$, respectively. The integral in (1) exists provided that *X* and *Y* have finite absolute moments of α-order. Write ${\mathrm{dCov}}^{2(\alpha )}\left(X\right)={\mathrm{dCov}}^{2(\alpha )}\left(X,X\right)$ and ${\mathrm{dCov}}^{2(\alpha )}\left(Y\right)={\mathrm{dCov}}^{2(\alpha )}\left(Y,Y\right)$. Analogously to Pearson correlation, distance correlation (dCor) is the normalized version of distance covariance, which is defined as

$$\begin{array}{c} {\mathrm{dCor}}^{2(\alpha )}\left(X,Y\right)=\left\{\begin{array}{cc} \frac{{\mathrm{dCov}}^{2(\alpha )}\left(X,Y\right)}{\sqrt{{\mathrm{dCov}}^{2(\alpha )}\left(X\right){\mathrm{dCov}}^{2(\alpha )}\left(Y\right)}},\; & \mathrm{if}\;\;{\mathrm{dCov}}^{2(\alpha )}\left(X\right){\mathrm{dCov}}^{2(\alpha )}\left(Y\right)>0,\\ 0,\; & \mathrm{if}\;\;{\mathrm{dCov}}^{2(\alpha )}\left(X\right){\mathrm{dCov}}^{2(\alpha )}\left(Y\right)=0. \end{array}\right. \end{array}$$

Székely et al. [1] showed that, for distributions with finite first moments, we have $0\leq {\mathrm{dCor}}^{2(\alpha )}\left(X,Y\right)\leq 1$, and ${\mathrm{dCor}}^{2(\alpha )}\left(X,Y\right)=0$ if and only if *X* and *Y* are mutually independent. Hence, distance correlation provides a natural extension of Pearson correlation and rank correlation in capturing arbitrary types of dependence. The extension of distance covariance from pairs of random variables to *n*-tuplets of random variables was addressed in Böttcher et al. [10]. Interested readers are referred to Székely et al. [1], Székely and Rizzo [2] and Böttcher et al. [10] for more details of the background of the distance covariance.

Uncorrelatedness is quite weak in comparison with independence. So, we are faced with this question: is there a concept that is stronger than uncorrelatedness but weaker than independence? The answer to this question is yes and it is called sub-independence. This concept was first introduced by Durairajan [11] and subsequently used in many papers; see, e.g., Hamedani and Volkmer [12], Ebrahimi et al. [13], Hamedani [14], Schennach [15], Dong et al. [16], and references therein. Sub-independence, although less intuitive than independence in appearance, provides a more flexible and realistic theoretical foundation in many practical modeling scenarios. Since it only requires that the distribution of the sum of variables can be calculated through convolution, rather than requiring that the complete joint distribution can be decomposed into a product form, it has specific applications in multiple fields. However, there are no indices to measure this dependence, such as the product moment covariance and correlation measuring the linear dependence of random variables and the distance covariance and distance correlation measuring all types of dependencies between random variables. Inspired by the groundbreaking work on distance covariance in Székely et al. [1], this paper focuses on a new dependence coefficient that measures the dependence between two random vectors *X* and *Y* of equal dimension. The main features of our coefficient are the following: it has a simple expression; it belongs to the range of [0, 1] and the value zero only if the random vectors are sub-independent; it is fully nonparametric; although our measure is based on the characteristic function, it can be simplified as an expression of moments. We study the properties, both theoretical and empirical, of the new coefficient. As applications, we present several examples, such as the concentration inequalities used in machine learning, renewal theorems and sums of randomly indexed sequences in the framework of sub-independence.

The rest of this paper is organized as follows. In Section 2, we propose a new measure, and the theoretical properties are discussed. In Section 3, the comparisons of sub-independence with known dependencies are investigated, and results for the bivariate normal and Cauchy are given in Section 4. Estimation, asymptotic properties, simulation studies and comparisons with other dependence measures are presented in Section 5, some applications and a real-data example are given in Section 6, and Section 7 gives the conclusions and prospects.

## 2. The New Class of Measures

The following notation is used throughout. The Euclidean norm for random vectors $X={\left(X_{1},\cdots ,X_{d}\right)}^{T}\in R^{d},d\geq 1$, is ${\left|X\right|}_{d}={\left(X_{1}^{2}+\cdots +X_{d}^{2}\right)}^{1/2}$, and 〈u,v〉 is the standard Euclidean scalar product of *u* and *v*. Whenever the dimension *d* is clear in context, we omit the subscript. The symbol |·| denotes the complex norm when its argument is complex; otherwise, |·| denotes the Euclidean norm. We denote by the imaginary unit $i=\sqrt{-1}$. Let $\phi_{X,Y}\left(s,t\right)=E\left(e^{i\langle s,X\rangle +i\langle t,Y\rangle }\right)$ be the joint characteristic function of $\left(X,Y\right)\in R^{p\times q},$ $\phi_{X}\left(s\right)=\phi_{X,Y}\left(s,0\right)$ and $\phi_{Y}\left(t\right)=\phi_{X,Y}\left(0,t\right)$ be the corresponding marginal characteristic functions. For any function ϕ, we denote ${\left|\phi \right|}^{2}=\phi \bar{\phi }$, where $\bar{\phi }$ denotes the complex conjugate of ϕ.

**Definition** **1** (Ebrahimi et al. [13], Schennach [15])**.** *(i) n real valued random variables $X_{1},\cdots ,X_{n}$ are said to be sub-independent if $\phi_{\sum_{i=1}^{n}X_{i}}\left(t\right)=\prod_{i=1}^{n}\phi_{X_{i}}\left(t\right)$ for all t∈R.*

*(ii) Random variables $X_{1},\cdots ,X_{n}$ are said to mutually sub-independent if, for each subset $\left\{X_{i_{1}},\cdots ,X_{i_{k}}\right\}$ (2≤k≤n) of $\left\{X_{1},\cdots ,X_{n}\right\}$, $\phi_{X_{i1}+\cdots +X_{ik}}\left(t\right)=\phi_{X_{i1},\cdots ,X_{ik}}\left(t,\cdots ,t\right)=\prod_{r=1}^{k}\phi_{X_{ir}}\left(t\right)$ for all t∈R.*

Alternatively, in terms of distributions, random variables $X_{i},i=1,2,\cdots ,n$ are sub-independent if the distribution of $\sum_{i=1}^{n}X_{i}$ is the convolution of the distributions of $X_{i}$’s. In this case, $X_{i}$’s are said to be summable uncorrelated marginals (SUMs). It is clear that the SUM and sub-independence are equivalent, so the two terminologies can be used interchangeably (cf. Ebrahimi et al. [13]). The concept of sub-independence defined above can be extended to random vectors of an arbitrary common dimension.

Although sub-independence is a weaker and more flexible assumption than full independence, to date, no statistical index has been available to measure this type of dependence. This paper fills that gap by proposing two new measures.

**Definition** **2.** *Let ${\left(X_{i}\right)}_{i=1,...,n}$ be random vectors with values in $R^{p}$. The α-sub-independent (0<α<2) covariance between random vectors $X_{1},\cdots ,X_{n}$ is the non-negative number $siCov^{\left(\alpha \right)}\left(X_{1},\cdots ,X_{n}\right)$ defined by*

(2) $$\begin{array}{c} siCov^{\left(\alpha \right)}\left(X_{1},\cdots ,X_{n}\right)=\int_{R^{p}}{\left|\phi_{X_{1},\cdots ,X_{n}}\left(t,\cdots ,t\right)-\prod_{i=1}^{n}\phi_{X_{i}}\left(t\right)\right|}^{2}\rho_{\alpha }\left(t\right)dt, \end{array}$$

*where $\rho_{\alpha }\left(t\right)=c{\left(p,\alpha \right)}^{-1}{\left|t\right|}_{p}^{-\alpha -p}.$ The α-sub-independent correlation between random vectors $X_{1},\cdots ,X_{n}$ is the non-negative number $siCor^{\left(\alpha \right)}\left(X_{1},\cdots ,X_{n}\right)$ defined by*

(3) $$\begin{array}{c} siCor^{\left(\alpha \right)}\left(X_{1},\cdots ,X_{n}\right)=\frac{siCov^{\left(\alpha \right)}\left(X_{1},\cdots ,X_{n}\right)}{\int_{R^{p}}\prod_{i=1}^{n}\left(1-\right|\phi_{X_{i}}\left(t\right){|}^{2})\rho_{\alpha }\left(t\right)dt}, \end{array}$$

*if $\int_{R^{p}}\prod_{i=1}^{n}(1-{\left|\phi_{X_{i}}\left(t\right)\right|}^{2})\rho_{\alpha }\left(t\right)dt>0$, or 0 otherwise.*

For simplicity, we restrict our discussion to the case n=2 in what follows. We denote 1-sub-independent covariance as sub-independent covariance, 1-sub-independent correlation as sub-independent correlation; we write $siCov^{\left(\alpha \right)}\left(X\right)=siCov^{\left(\alpha \right)}\left(X,X\right)$, $siCov^{\left(\alpha \right)}\left(Y\right)$$=siCov^{\left(\alpha \right)}\left(Y,Y\right)$, $siCov\left(X,Y\right)=siCov^{\left(1\right)}\left(X,Y\right)$ and $siCor\left(X,Y\right)=siCor^{\left(1\right)}\left(X,Y\right)$.

**Remark** **1.** *Condition $\int_{R^{p}}\left(1-{\left|\phi_{X}\left(t\right)\right|}^{2}\right)\left(1-{\left|\phi_{Y}\left(t\right)\right|}^{2}\right)\rho_{\alpha }\left(t\right)dt>0$ is equivalent to neither X nor Y being a constant vector. Unlike the normalized version of distance covariance in Székely et al. [1], $siCor^{\left(\alpha \right)}\left(X,Y\right)$ cannot be defined in the form of*

$$siCor^{\left(\alpha \right)}\left(X,Y\right)=\frac{siCov^{\left(\alpha \right)}\left(X,Y\right)}{\sqrt{siCov^{\left(\alpha \right)}\left(X\right)\cdot siCov^{\left(\alpha \right)}\left(Y\right)}},$$

*which violates the usual condition that $siCor^{\left(\alpha \right)}\left(X,Y\right)\leq 1$; see a counterexample in Remark 2.*

For the finiteness of $siCov^{\left(\alpha \right)}\left(X,Y\right)$, it is sufficient that $E(|X{|}_{p}^{\alpha }+|Y{|}_{p}^{\alpha })<\infty$. The following lemma is crucial, and can be found in Székely and Rizzo [2]; see also von Bahr and Esseen [17] for the case p=1.

**Lemma** **1.** *Assume that 0<α<2 and $X\in R^{p}$; if ${E|X|}_{p}^{\alpha }<\infty$, then*

(4) $$\begin{array}{c} \int_{R^{p}}\frac{1-Ee^{i\langle t,X\rangle }}{{\left|t\right|}_{p}^{\alpha +p}}dt=\int_{R^{p}}\frac{1-E\cos\langle t,X\rangle }{{\left|t\right|}_{p}^{\alpha +p}}dt=c\left(p,\alpha \right)E{\left|X\right|}_{p}^{\alpha }. \end{array}$$

To prove $siCov^{\left(\alpha \right)}\left(X,Y\right)<\infty$, by the Cauchy–Schwarz inequality, we have

$$\begin{array}{ccc} {\left|\phi_{X,Y}\left(t,t\right)-\phi_{X}\left(t\right)\phi_{Y}\left(t\right)\right|}^{2} & = & {\left|E\left(e^{i<t,X>}-\phi_{X}\left(t\right)\right)\left(e^{i<t,Y>}-\phi_{Y}\left(t\right)\right)\right|}^{2}\\ & \leq & {\left(E[|e^{i<t,X>}-\phi_{X}\left(t\right)||e^{i<t,Y>}-\phi_{Y}\left(t\right)|]\right)}^{2}\\ & \leq & E{\left|e^{i<t,X>}-\phi_{X}\left(t\right)\right|}^{2}E{\left|e^{i<t,Y>}-\phi_{Y}\left(t\right)\right|}^{2}\\ & = & (1-{\left|\phi_{X}\left(t\right)\right|}^{2})(1-{\left|\phi_{Y}\left(t\right)\right|}^{2}). \end{array}$$

Then, by Lemma 1 and by Fubini’s theorem, it follows that

$$\begin{array}{ccc} {\mathrm{siCov}}^{\left(\alpha \right)}\left(X,Y\right) & = & \int_{R^{p}}{\left|\phi_{X,Y}\left(t,t\right)-\phi_{X}\left(t\right)\phi_{Y}\left(t\right)\right|}^{2}\rho_{\alpha }\left(t\right)dt\\ & \leq & \int_{R^{p}}\left(1-{\left|\phi_{X}\left(t\right)\right|}^{2}\right)\left(1-{\left|\phi_{Y}\left(t\right)\right|}^{2}\right)\rho_{\alpha }\left(t\right)dt\\ & \leq & \sqrt{\int_{R^{p}}{\left(1-{\left|\phi_{X}\left(t\right)\right|}^{2}\right)}^{2}\rho_{\alpha }\left(t\right)dt\int_{R^{p}}{\left(1-{\left|\phi_{Y}\left(t\right)\right|}^{2}\right)}^{2}\rho_{\alpha }\left(t\right)dt}\\ & \leq & \sqrt{\int_{R^{p}}\left(1-{\left|\phi_{X}\left(t\right)\right|}^{2}\right)\rho_{\alpha }\left(t\right)dt\int_{R^{p}}\left(1-{\left|\phi_{Y}\left(t\right)\right|}^{2}\right)\rho_{\alpha }\left(t\right)dt}\\ & = & \sqrt{E{\left|X-X^{\prime }\right|}_{p}^{\alpha }E{\left|Y-Y^{\prime }\right|}_{p}^{\alpha }}<\infty . \end{array}$$

Similarly,

$$\begin{array}{ccc} {\mathrm{siCov}}^{\left(\alpha \right)}\left(X\right) & = & \int_{R^{p}}{\left|\phi_{X,X}\left(t,t\right)-\phi_{X}\left(t\right)\phi_{X}\left(t\right)\right|}^{2}\rho_{\alpha }\left(t\right)dt\\ & \leq & \int_{R^{p}}{\left(E{\left|e^{i<t,X>}-\phi_{X}\left(t\right)\right|}^{2}\right)}^{2}\rho_{\alpha }\left(t\right)dt\\ & = & \int_{R^{p}}{\left(E\left(e^{i<t,X>}-\phi_{X}\left(t\right)\right)\left(e^{-i<t,X>}-\phi_{X}\left(-t\right)\right)\right)}^{2}\rho_{\alpha }\left(t\right)dt\\ & = & \int_{R^{p}}{\left(1-Ee^{i<t,X>}Ee^{-i<t,X>}\right)}^{2}\rho_{\alpha }\left(t\right)dt\\ & = & \int_{R^{p}}{\left(1-{\left|\phi_{X}\left(t\right)\right|}^{2}\right)}^{2}\rho_{\alpha }\left(t\right)dt\\ & \leq & \int_{R^{p}}\left(1-{\left|\phi_{X}\left(t\right)\right|}^{2}\right)\rho_{\alpha }\left(t\right)dt\\ & = & E{\left|X-X^{\prime }\right|}_{p}^{\alpha }<\infty , \end{array}$$

and

$$\begin{array}{c} {\mathrm{siCov}}^{\left(\alpha \right)}\left(Y\right)=\int_{R^{p}}{\left|\phi_{Y,Y}\left(t,t\right)-\phi_{Y}\left(t\right)\phi_{Y}\left(t\right)\right|}^{2}\rho_{\alpha }\left(t\right)dt\leq E{\left|Y-Y^{\prime }\right|}_{p}^{\alpha }<\infty . \end{array}$$

The following results summarize the key properties of sub-independent covariance.

**Theorem** **1.** *The following properties hold:*

*(1). For all $X,Y\in R^{p}$, $siCov^{\left(\alpha \right)}\left(X,Y\right)\geq 0$, and $siCov^{\left(\alpha \right)}\left(X,Y\right)=0$ if and only if X and Y are sub-independent.*

*(2). For all $X\in R^{p}$, $siCov^{\left(\alpha \right)}\left(X\right)=0$ iff X=E(X), almost surely.*

*(3). $siCov^{\left(\alpha \right)}\left(a_{1}+bCX,a_{2}+bCY\right)={\left|b\right|}^{\alpha }siCov^{\left(\alpha \right)}\left(X,Y\right)$, for all constant vectors $a_{1},a_{2}\in R^{p}$, scalar b and p×p orthonormal matrix C.*

*(4). $siCov^{\left(\alpha \right)}\left(a+bCX\right)={\left|b\right|}^{\alpha }siCov^{\left(\alpha \right)}\left(X\right)$, for all constant vector $a\in R^{p}$, scalar b and orthonormal matrix $C\in R^{p}$.*

*(5). If random variables X and Y are sub-independent, then for any α>0 such that $E(|X{|}^{\alpha }+|Y{|}^{\alpha })<\infty$, one has $siCov^{\left(\alpha \right)}\left(X,Y\right)=0$. Conversely, if $siCov^{\left(\alpha \right)}\left(X,Y\right)=0$ for some α>0, then X and Y are sub-independent.*

**Proof.** 

(1) If *X* and *Y* are sub-independent, then $\phi_{X,Y}\left(t,t\right)=\phi_{X}\left(t\right)\phi_{Y}\left(t\right)$ for all $t\in R^{p}$; thus, $siCov^{\left(\alpha \right)}\left(X,Y\right)=0$. Conversely, if *X* and *Y* are not sub-independent, then there exists an open set $B\subset R^{p}$ such that $|\phi_{X,Y}\left(t,t\right)-\phi_{X}\left(t\right)\phi_{Y}{\left(t\right)|}^{2}>0$ for all t∈B, since characteristic functions are continuous. Hence, $siCov^{\left(\alpha \right)}\left(X,Y\right)>0$.
(2) $siCov^{\left(\alpha \right)}\left(X\right)=0$ iff $\phi_{X,X}\left(t,t\right)=\phi_{X}\left(t\right)\phi_{X}\left(t\right)$ for all $t\in R^{p}$; hence, *X* is a constant vector EX, almost surely.
(3) For all constant vectors $a_{1},a_{2}\in R^{p}$, scalar *b* and p×p orthonormal matrix *C*, we have
  $$\begin{array}{ccc} & & siCov^{\left(\alpha \right)}\left(a_{1}+bCX,a_{2}+bCY\right)\\ & = & \int_{R^{p}}{\left|Ee^{it^{\prime }\left(bCX+bCY\right)}-Ee^{it^{\prime }\left(bCX\right)}Ee^{it^{\prime }\left(bCY\right)}\right|}^{2}\rho_{\alpha }\left(t\right)dt\\ & = & \int_{R^{p}}{\left|Ee^{i{\left(bC^{\prime }t\right)}^{\prime }\left(X+Y\right)}-Ee^{i{\left(bC^{\prime }t\right)}^{\prime }X}Ee^{i{\left(bC^{\prime }t\right)}^{\prime }Y}\right|}^{2}\rho_{\alpha }\left(t\right)dt\\ & = & {\left|b\right|}^{\alpha }\int_{R^{p}}{\left|Ee^{is^{\prime }\left(X+Y\right)}-Ee^{is^{\prime }X}Ee^{is^{\prime }Y}\right|}^{2}\rho_{\alpha }\left(s\right)ds\\ & = & {\left|b\right|}^{\alpha }siCov^{\left(\alpha \right)}\left(X,Y\right), \end{array}$$
  where, in the last equality, we have used the transformation $s=bC^{\prime }t$ and the homogeneity relation $\rho_{\alpha }\left(t\right)dt={\left|b\right|}^{\alpha }\rho_{\alpha }\left(s\right)ds$.
(4) This is a special case of (3) for $a_{1}=a_{2}$ and Y=X. Statement (5) is obvious.

□

Analogous properties to the product–moment correlation coefficient for sub-independent correlation are established in Theorem 2.

**Theorem** **2.** *If X,Y are $R^{p}$-valued random variables, and ${E|X|}_{p}^{\alpha }+E{\left|Y\right|}_{p}^{\alpha }<\infty$, then the following properties hold:*

*(i)*  *$0\leq siCor^{\left(\alpha \right)}\left(X,Y\right)\leq 1$.*
*(ii)*  *If $siCor^{\left(\alpha \right)}\left(X,Y\right)=0$ for some α, then X and Y are sub-independent; Conversely, if X and Y are sub-independent, then for any α>0, we have $siCor^{\left(\alpha \right)}\left(X,Y\right)=0$.*
*(iii)*  *$siCor^{\left(\alpha \right)}\left(a_{1}+bCX,a_{2}+bCY\right)=1$ for all constant vectors $a_{1},a_{2}\in R^{p}$, scalar b and p×p orthonormal matrix C.*

**Proof.**  The statement $siCor^{\left(\alpha \right)}\left(X,Y\right)\geq 0$ is obvious. The only thing to show is that $siCor^{\left(\alpha \right)}\left(X,Y\right)\leq 1$. We just note that

$$\begin{array}{ccc} siCov^{\left(\alpha \right)}\left(X,Y\right) & = & \int_{R^{p}}{\left|\phi_{X,Y}\left(t,t\right)-\phi_{X}\left(t\right)\phi_{Y}\left(t\right)\right|}^{2}\rho_{\alpha }\left(t\right)dt\\ & \leq & \int_{R^{p}}\left(1-{\left|\phi_{X}\left(t\right)\right|}^{2}\right)\left(1-{\left|\phi_{Y}\left(t\right)\right|}^{2}\right)\rho_{\alpha }\left(t\right)dt, \end{array}$$

this proves (i). Statement (ii) is obvious.

(iii) This follows from Theorem 1 (iii) and (iv). □

**Theorem** **3.** *Suppose that $\left(X_{1},Y_{1}\right),\cdots ,\left(X_{4},Y_{4}\right)$ are independent copies of $\left(X,Y\right)\in R^{p\times p}$. If ${E|X|}_{p}^{\alpha }+E{\left|Y\right|}_{p}^{\alpha }<\infty$, then*

(5) $$\begin{array}{ccc} siCov^{\left(\alpha \right)}\left(X,Y\right) & = & 2E{\left|X_{1}+Y_{1}-X_{2}-Y_{3}\right|}_{p}^{\alpha }-E{\left|X_{1}+Y_{2}-X_{3}-Y_{4}\right|}_{p}^{\alpha }\\ & & -E{\left|X_{1}+Y_{1}-X_{2}-Y_{2}\right|}_{p}^{\alpha }, \end{array}$$

$$\begin{array}{ccc} siCov^{\left(\alpha \right)}\left(X\right) & = & 2E{\left|2X_{1}-X_{2}-X_{3}\right|}_{p}^{\alpha }-E{\left|X_{1}+X_{2}-X_{3}-X_{4}\right|}_{p}^{\alpha }\\ & & -2^{\alpha }E{\left|X_{1}-X_{2}\right|}_{p}^{\alpha }, \end{array}$$

*and*

$$\begin{array}{ccc} siCov^{\left(\alpha \right)}\left(Y\right) & = & 2E{\left|2Y_{1}-Y_{2}-Y_{3}\right|}_{p}^{\alpha }-E{\left|Y_{1}+Y_{2}-Y_{3}-Y_{4}\right|}_{p}^{\alpha }\\ & & -2^{\alpha }E{\left|Y_{1}-Y_{2}\right|}_{p}^{\alpha }. \end{array}$$

**Proof.**  We provide the proof for (5); the proofs of other parts are similar. Clearly,

$$\begin{array}{ccc} \phi_{X,Y}\left(t,t\right)\bar{\phi_{X,Y}\left(t,t\right)} & = & Ee^{it^{\prime }\left(X+Y\right)}Ee^{-it^{\prime }\left(X+Y\right)}\\ & = & Ee^{it^{\prime }\left(X+Y\right)}Ee^{-it^{\prime }\left(X_{2}+Y_{2}\right)}\\ & = & Ee^{it^{\prime }\left(X_{1}+Y_{1}-X_{2}-Y_{2}\right)}, \end{array}$$

$$\begin{array}{ccc} \phi_{X,Y}\left(t,t\right)\bar{\phi_{X}\left(t\right)\phi_{Y}\left(t\right)} & = & Ee^{it^{\prime }\left(X+Y\right)}Ee^{-it^{\prime }\left(X_{2}+Y_{3}\right)}\\ & = & Ee^{it^{\prime }\left(X_{1}+Y_{1}-X_{2}-Y_{3}\right)}, \end{array}$$

$$\begin{array}{ccc} \bar{\phi_{X,Y}\left(t,t\right)}\phi_{X}\left(t\right)\phi_{Y}\left(t\right) & = & Ee^{-it^{\prime }\left(X+Y\right)}Ee^{it^{\prime }\left(X_{2}+Y_{3}\right)}\\ & = & Ee^{-it^{\prime }\left(X_{1}+Y_{1}-X_{2}-Y_{3}\right)}, \end{array}$$

$$\begin{array}{ccc} \phi_{X}\left(t\right)\phi_{Y}\left(t\right)\bar{\phi_{X}\left(t\right)\phi_{Y}\left(t\right)} & = & Ee^{it^{\prime }\left(X_{1}+Y_{2}\right)}Ee^{-it^{\prime }\left(X_{3}+Y_{4}\right)}\\ & = & Ee^{it^{\prime }\left(X_{1}+Y_{2}-X_{3}-Y_{4}\right)}. \end{array}$$

Then, by applying Lemma 1, we obtain

$$\begin{array}{ccc} siCov^{\left(\alpha \right)}\left(X,Y\right) & = & \int_{R^{p}}\left(\phi_{X,Y}\left(t,t\right)-\phi_{X}\left(t\right)\phi_{Y}\left(t\right)\right)\bar{\left(\phi_{X,Y}\left(t,t\right)-\phi_{X}\left(t\right)\phi_{Y}\left(t\right)\right)}\rho_{\alpha }\left(t\right)dt\\ & = & -\int_{R^{p}}\left[\phi_{X,Y}\left(t,t\right)\bar{\phi_{X}\left(t\right)\phi_{Y}\left(t\right)}+\bar{\phi_{X,Y}\left(t,t\right)}\phi_{X}\left(t\right)\phi_{Y}\left(t\right)\right]\rho_{\alpha }\left(t\right)dt\\ & & +\int_{R}\left[\phi_{X}\left(t\right)\phi_{Y}\left(t\right)\bar{\phi_{X}\left(t\right)\phi_{Y}\left(t\right)}+\phi_{X,Y}\left(t,t\right)\bar{\phi_{X,Y}\left(t,t\right)}\right]\rho_{\alpha }\left(t\right)dt\\ & = & \int_{R^{p}}\left(1-\phi_{X,Y}\left(t,t\right)\bar{\phi_{X}\left(t\right)\phi_{Y}\left(t\right)}\right)\rho_{\alpha }\left(t\right)dt\\ & & +\int_{R^{p}}\left(1-\bar{\phi_{X,Y}\left(t,t\right)}\phi_{X}\left(t\right)\phi_{Y}\left(t\right)\right)\rho_{\alpha }\left(t\right)dt\\ & & -\int_{R^{p}}\left(1-\phi_{X}\left(t\right)\phi_{Y}\left(t\right)\bar{\phi_{X}\left(t\right)\phi_{Y}\left(t\right)}\right)\rho_{\alpha }\left(t\right)dt\\ & & -\int_{R^{p}}\left(1-\phi_{X,Y}\left(t,t\right)\bar{\phi_{X,Y}\left(t,t\right)}\right)\rho_{\alpha }\left(t\right)dt\\ & = & \int_{R^{p}}\left(1-Ee^{it^{\prime }\left(X_{1}+Y_{1}-X_{2}-Y_{3}\right)}\right)\rho_{\alpha }\left(t\right)dt\\ & & +\int_{R^{p}}\left(1-Ee^{-it^{\prime }\left(X_{1}+Y_{1}-X_{2}-Y_{3}\right)}\right)\rho_{\alpha }\left(t\right)dt\\ & & -\int_{R^{p}}\left(1-Ee^{it^{\prime }\left(X_{1}+Y_{2}-X_{3}-Y_{4}\right)}\right)\rho_{\alpha }\left(t\right)dt\\ & & -\int_{R^{p}}\left(1-Ee^{it^{\prime }\left(X_{1}+Y_{1}-X_{2}-Y_{2}\right)}\right)\rho_{\alpha }\left(t\right)dt\\ & = & 2E{\left|X_{1}+Y_{1}-X_{2}-Y_{3}\right|}_{p}^{\alpha }-E{\left|X_{1}+Y_{2}-X_{3}-Y_{4}\right|}_{p}^{\alpha }\\ & & -E{\left|X_{1}+Y_{1}-X_{2}-Y_{2}\right|}_{p}^{\alpha }. \end{array}$$

This completes the proof. □

The next theorem gives a equivalent representation of siCor(X,Y).

**Theorem** **4.** *Suppose that $\left(X_{1},Y_{1}\right),\cdots ,\left(X_{4},Y_{4}\right)$ are independent, identically distributed copies of $\left(X,Y\right)\in R^{p\times p}$; if $E(|X{|}_{p}^{\alpha }+|Y{|}_{p}^{\alpha })<\infty$, then*

(6) $$\begin{array}{c} siCor^{\left(\alpha \right)}\left(X,Y\right)=\frac{2J_{1}\left(X,Y\right)-J_{2}\left(X,Y\right)-J_{3}\left(X,Y\right)}{K_{1}\left(X,Y\right)+K_{2}\left(X,Y\right)-K_{3}\left(X,Y\right)}, \end{array}$$

*where*

$$\begin{array}{ccc} J_{1}\left(X,Y\right) & = & E|X_{1}+Y_{1}-X_{2}-Y_{3}{|}_{p}^{\alpha },\\ J_{2}\left(X,Y\right) & = & E|X_{1}+Y_{2}-X_{3}-Y_{4}{|}_{p}^{\alpha },\\ J_{3}\left(X,Y\right) & = & E|X_{1}+Y_{1}-X_{2}-Y_{2}{|}_{p}^{\alpha }, \end{array}$$

*and*

$$\begin{array}{ccc} K_{1}\left(X,Y\right) & = & E|X_{1}-X_{2}{|}_{p}^{\alpha },\\ K_{2}\left(X,Y\right) & = & E|Y_{3}-Y_{4}{|}_{p}^{\alpha },\\ K_{3}\left(X,Y\right) & = & E|X_{1}-X_{2}+Y_{3}-Y_{4}{|}_{p}^{\alpha }. \end{array}$$

**Proof.**  $J_{1}\left(X,Y\right),J_{2}\left(X,Y\right),$ and $J_{3}\left(X,Y\right)$ can be obtained by (5). For the convenience of writing, if we set $▵=\int_{R^{p}}\left(1-{\left|\phi_{X}\left(t\right)\right|}^{2}\right)\left(1-{\left|\phi_{Y}\left(t\right)\right|}^{2}\right)\rho_{\alpha }\left(t\right)dt$, then

$$\begin{array}{ccc} ▵ & = & \int_{R^{p}}\left(1-e^{it(X_{1}-X_{2})}\right)\left(1-e^{it(Y_{3}-Y_{4})}\right)\rho_{\alpha }\left(t\right)dt\\ & = & \int_{R^{p}}\left(1-e^{it(X_{1}-X_{2})}\right)\rho_{\alpha }\left(t\right)dt\\ & & +\int_{R^{p}}\left(1-e^{it(Y_{3}-Y_{4})}\right)\rho_{\alpha }\left(t\right)dt\\ & & -\int_{R^{p}}\left(1-e^{it(X_{1}-X_{2}+Y_{3}-Y_{4})}\right)\rho_{\alpha }\left(t\right)dt\\ & = & E{\left|X_{1}-X_{2}\right|}_{p}^{\alpha }+E{\left|Y_{3}-Y_{4}\right|}_{p}^{\alpha }-E{\left|X_{1}-X_{2}+Y_{3}-Y_{4}\right|}_{p}^{\alpha }\\ & = & K_{1}\left(X,Y\right)+K_{2}\left(X,Y\right)-K_{3}\left(X,Y\right). \end{array}$$

Hence, the theorem. □

## 3. Comparisons with Known Dependencies

The classical Pearson product–moment correlations between two variables have been generalized to measure association between two variables in many ways, for example, the distance covariance (dCov) coefficient, martingale difference correlation (Shao and Zhang [4]) and sub-independent coefficient. A large body of the literature uses these coefficients to test whether two random vectors are correlated with each other. Accordingly, it is important to clarify the logical relationships among these notions of dependence. We discuss the relationship among Pearson product–moment correlation, distance covariance coefficient, sub-independent coefficient and positive orthant dependence (POD) and negative orthant dependence (NOD) dependence. For details about POD (NOD) and other notions of dependence, see, e.g., Barlow and Proschan [18].

Ebrahimi et al. [13] pointed out that POD (NOD) is the weakest among all existing notions of dependence. We remark that, for the sub-independence and POD (NOD), there is no logical implication relationship. To see that, let $X=(X_{1},\cdots ,X_{n})$ be a non-negative random vector in $R^{n}$; then, for t≥0,

$$\begin{array}{ccc} \phi_{\sum_{k=1}^{n}X_{k}}\left(t\right) & - & \prod_{i=1}^{n}\phi_{X_{i}}\left(t\right)\\ & = & \int_{0}^{\infty }\cdots \int_{0}^{\infty }e^{-t\sum_{i=1}^{n}x_{i}}\left(\bar{F}\left(x_{1},\cdots ,x_{n}\right)-\prod_{i=1}^{n}\bar{F_{i}}\left(x_{i}\right)\right)dx_{1}\cdots x_{n}, \end{array}$$

from which we cannot conclude that there is a mutual inclusion relationship between POD and sub-independence. Namely, there exist random variable pairs that satisfy POD but do not satisfy sub-independence (for example, variables that are strongly positively correlated but the distribution of their sum is not in a convolutional form). There are also cases where sub-independence is satisfied but POD is not (for example, the distribution of the sum conforms to convolution, but the tail exhibits negative dependence). In the SUM distribution class, POD+sub-independence ⇒ independence (see, e.g., Lemma 4 in [13]).

For rvs *U* and *V*, Shao and Zhang [4] proposed a martingale difference correlation for high-dimensional variable screening; they called V−E(V) a martingale difference with respect to *U* if E(V|U)=E(V). This relationship lies in between independence and uncorrelatedness, and neither this relation nor sub-independence implies the other. Namely, the martingale difference correlation and sub-independence have no inclusion relationship in the mathematical framework.

Edelmann et al. [19] asserted that “dCor(X,Y)=1 if and only if *Y* is a linear function of *X*, almost surely”. Edelmann et al. [20] also asserted that “|Cor(X,Y)|=1 if and only if ${\mathrm{dCor}}^{2}\left(X,Y\right)=1$”.

The following result shows that the sub-independence lies in between independence and uncorrelatedness.

**Theorem** **5.** *If X,Y are real-valued random variables, and $EX^{2}+EY^{2}<\infty$, then*

*(i)*  *${\mathrm{dCor}}^{2}\left(X,Y\right)=0\Rightarrow siCor\left(X,Y\right)=0\Rightarrow \mathrm{Cor}\left(X,Y\right)=0,$ but the converse is not true.*
*(ii)*  *$\mathrm{Cor}\left(X,Y\right)\neq 0\Rightarrow siCor\left(X,Y\right)\neq 0\Rightarrow {\mathrm{dCor}}^{2}\left(X,Y\right)\neq 0$.*
*(iii)*  *If Y=X+a for constant a, then siCor(X,Y)=1.*

**Proof.**  (i) The proof is straightforward. In fact, ${\mathrm{dCor}}^{2}\left(X,Y\right)=0\Leftrightarrow \phi_{X,Y}\left(s,t\right)=\phi_{X}\left(s\right)\phi_{Y}\left(t\right)$ for all s,t∈R$\Rightarrow \phi_{X,Y}\left(t,t\right)=\phi_{X}\left(t\right)\phi_{Y}\left(t\right)$ for all t∈R⇒E(XY)=E(X)E(Y)⇔Cor(X,Y)=0. The example in the next section illustrates that the converse of (i) is not true.

It is not difficult to find examples from standard textbooks that there are random vectors such that Cor(X,Y)=0 holds but siCor(X,Y)=0 is violated, or siCor(X,Y)=0 holds but ${\mathrm{dCor}}^{2}\left(X,Y\right)=0$ is not satisfied.

Statement (ii) is easily obtained by (i). □

## 4. Two Examples

This section provides siCov(X,Y) and siCor(X,Y) for two-dimensional normal distribution and two-dimensional Cauchy distribution.

### 4.1. Results for the Bivariate Normal Distribution

Let (X,Y) have the bivariate standard normal distribution $N_{2}\left(0,0,1,1,\rho \right)$. Suppose that $\left(X_{1},Y_{1}\right),\cdots ,\left(X_{4},Y_{4}\right)$ are independent, identically distributed copies of (X,Y); then,

$$\begin{array}{ccc} & & X_{1}+Y_{1}-X_{2}-Y_{3}∼N\left(0,4+2\rho \right),\;X_{1}+Y_{2}-X_{3}-Y_{4}∼N\left(0,4\right),\\ & & X_{1}+Y_{1}-X_{2}-Y_{2}∼N\left(0,4\left(1+\rho \right)\right), \end{array}$$

and hence

$$\begin{array}{ccc} & & E|X_{1}+Y_{1}-X_{2}-Y_{3}|=\sqrt{4+2\rho }\sqrt{\frac{2}{\pi }},\;E\left|X_{1}+Y_{2}-X_{3}-Y_{4}\right|=2\sqrt{\frac{2}{\pi }},\\ & & E|X_{1}+Y_{1}-X_{2}-Y_{2}|=2\sqrt{1+\rho }\sqrt{\frac{2}{\pi }}. \end{array}$$

Making use of Theorem 3 yields

$$\begin{array}{c} siCov\left(X,Y\right)=2\sqrt{4+2\rho }\sqrt{\frac{2}{\pi }}-2\sqrt{\frac{2}{\pi }}-2\sqrt{1+\rho }\sqrt{\frac{2}{\pi }}. \end{array}$$

Similarly,

$$\begin{array}{c} siCov\left(X,X\right)=siCov\left(Y,Y\right)=2\sqrt{\frac{2}{\pi }}\left(\sqrt{6}-\sqrt{2}-1\right), \end{array}$$

and

(7) $$K:=E|X_{1}-X_{2}\left|+E\right|Y_{3}-Y_{4}\left|-E\right|X_{1}-X_{2}+Y_{3}-Y_{4}|=2\sqrt{\frac{2}{\pi }}\left(\sqrt{2}-1\right).$$

Finally, by (6), we have

$$\begin{array}{c} siCor_{\rho }\left(X,Y\right):=siCor\left(X,Y\right)=\frac{\sqrt{4+2\rho }-\sqrt{1+\rho }-1}{\sqrt{2}-1}, \end{array}$$

from which we get siCor(X,Y)≤|ρ|, and

$$\begin{array}{c} siCor_{0}\left(X,Y\right)=0,\;siCor_{-1}\left(X,Y\right)=1,\;siCor_{1}\left(X,Y\right)=\frac{\sqrt{6}-\sqrt{2}-1}{\sqrt{2}-1}≐0.085. \end{array}$$

**Remark** **2.** *Define*

(8) $$R_{\rho }:=\frac{siCov(X,Y)}{\sqrt{siCov(X,X)\cdot siCov(Y,Y)}}.$$

*Then,*

(9) $$R_{\rho }=\frac{\sqrt{4+2\rho }-\sqrt{1+\rho }-1}{\sqrt{6}-\sqrt{2}-1}.$$

*In particular,*

$$R_{0}=0,\;R_{-1}=\frac{\sqrt{2}-1}{\sqrt{6}-\sqrt{2}-1}≐11.74,\;R_{1}=1.$$

*It turns out that $R_{\rho }$ cannot satisfy the desired condition $R_{\rho }\leq 1$.*

### 4.2. Results for the Bivariate Cauchy Distribution

The bivariate Cauchy distribution is a continuous probability distribution used to describe the joint behavior of two random variables, and is an important special case of multivariate heavy-tailed distributions. It has special significance in statistical modeling, especially suitable for scenarios with strong correlation between extreme events and where traditional normality assumptions do not hold. The two-dimensional random vector $X={\left(X_{1},X_{2}\right)}^{\prime }$ is said to have a multivariate Cauchy distribution (denoted by $X∼MC_{2}\left(\mu ,\Sigma \right)$) if its probability density function is given by [21]

$$\frac{1}{2\pi }{\left|\Sigma \right|}^{-\frac{1}{2}}{\left(1+{\left(x-\mu \right)}^{\prime }{\left|\Sigma \right|}^{-1}(x-\mu \right)}^{-\frac{3}{2}},\;x\in R^{2},$$

where $\mu \in R^{2}$ is its location parameter and Σ is a 2×2 positive semi-definite matrix. The characteristic function of $X∼MC_{2}\left(\mu ,\Sigma \right)$ is given by

$$\phi_{X}\left(t\right)=e^{it^{\prime }\mu }\exp\left(-\sqrt{t^{\prime }\Sigma t}\right),\;t\in R^{2}.$$

In particular, the standard multivariate Cauchy distribution $MC_{2}\left(0,I_{2}\right)$ has the density

$$\frac{1}{2\pi }{\left(1+x^{\prime }x\right)}^{-\frac{3}{2}},\;x\in R^{2},$$

and the characteristic function is given by

$$\phi_{\left(X_{1},X_{2}\right)}\left(t_{1},t_{2}\right)=\exp\left(-\sqrt{t_{1}^{2}+t_{2}^{2}}\right),\;-\infty <t_{1},t_{2}<\infty ,$$

It follows that

$$\phi_{X_{1}}\left(t\right)=\phi_{X_{2}}\left(t\right)=\exp\left(-|t|\right),\;-\infty <t<\infty ,$$

and

$$\phi_{X_{1}+X_{2}}\left(t\right)=\exp\left(-\sqrt{2}\left|t\right|\right),\;-\infty <t<\infty .$$

By (2), for 0<α<1,

$$\begin{array}{ccc} siCov^{\left(\alpha \right)}\left(X_{1},X_{2}\right) & = & \frac{1}{c(1,\alpha )}\int_{R}{\left|\exp\left(-\sqrt{2}\left|t\right|\right)-\exp\left(-2|t|\right)\right|}^{2}{\left|t\right|}^{-1-\alpha }dt\\ & = & \frac{2}{c(1,\alpha )}\int_{0}^{\infty }{\left|\exp\left(-\sqrt{2}t\right)-\exp\left(-2t\right)\right|}^{2}t^{-1-\alpha }dt\\ & = & \frac{2}{c(1,\alpha )}\int_{0}^{\infty }\left(e^{-4t}-2e^{-(2+\sqrt{2})t}+e^{-2\sqrt{2}t}\right)t^{-1-\alpha }dt\\ & = & \frac{2}{c(1,\alpha )\alpha }\left(-4^{\alpha }+2{\left(2+\sqrt{2}\right)}^{\alpha }-{\left(2\sqrt{2}\right)}^{\alpha }\right)\Gamma \left(1-\alpha \right), \end{array}$$

and

$$\begin{array}{ccc} \int_{R}\left(1-{\left|\phi_{X_{1}}\left(t\right)\right|}^{2}\right)\left(1-{\left|\phi_{X_{2}}\left(t\right)\right|}^{2}\right)\rho_{\alpha }\left(t\right)dt & = & \frac{1}{c(1,\alpha )}\int_{R}{\left(1-e^{-2|t|}\right)}^{2}{\left|t\right|}^{-1-\alpha }dt\\ & = & \frac{2}{c(1,\alpha )}\int_{0}^{\infty }{\left(1-e^{-2t}\right)}^{2}t^{-1-\alpha }dt\\ & = & \frac{8}{c(1,\alpha )\alpha }\left(2^{\alpha -1}-4^{\alpha -1}\right)\Gamma \left(1-\alpha \right). \end{array}$$

Finally, by (3), we obtain

(10) $$\begin{array}{c} siCor^{\left(\alpha \right)}\left(X,Y\right)=\frac{-4^{\alpha }+2{\left(2+\sqrt{2}\right)}^{\alpha }-{\left(2\sqrt{2}\right)}^{\alpha }}{4(2^{\alpha -1}-4^{\alpha -1})}. \end{array}$$

Define

$$l\left(\alpha \right)=\frac{-4^{\alpha }+2{\left(2+\sqrt{2}\right)}^{\alpha }-{\left(2\sqrt{2}\right)}^{\alpha }}{4\left(2^{\alpha -1}-4^{\alpha -1}\right)},\;\alpha \in \left(0,1\right).$$

After a lengthy but elementary analysis, one obtains $l^{\prime }\left(\alpha \right)>0$ for all α∈(0,1); hence, l(α) is strictly increasing on (0,1). Moreover,

(11) $$\lim_{\alpha \to 0^{+}}l\left(\alpha \right)=0,\;\lim_{\alpha \to 1^{-}}l\left(\alpha \right)=\frac{-2\ln4+2p\ln\left(2+\sqrt{2}\right)-\frac{3}{2}\ln\left(2\sqrt{2}\right)}{2\ln2}\approx 0.03643,$$

where $p=\log_{2}\left(2+\sqrt{2}\right)=\frac{\ln(2+\sqrt{2})}{\ln2}\approx 1.7716$. Hence, $0<siCor^{\left(\alpha \right)}\left(X,Y\right)<0.03643$.

## 5. Estimation and Asymptotic Properties

In this section, we give an unbiased estimate of $siCov^{\left(\alpha \right)}\left(X,Y\right)$ (denote by ${\hat{siCov^{\left(\alpha \right)}}}_{n}\left(X,Y\right)$), and derive the asymptotic distribution of ${\hat{siCov^{\left(\alpha \right)}}}_{n}\left(X,Y\right)$.

Define the symmetric kernel function

$$\begin{array}{ccc} k(\left(X_{1},Y_{1}\right),\left(X_{2},Y_{2}\right),\left(X_{3},Y_{3}\right),\left(X_{4},Y_{4}\right)) & = & \frac{1}{12}\sum_{\overset{1\leq i,j,k\leq 4}{i\neq j\neq k}}{\left|X_{i}+Y_{i}-X_{j}-Y_{k}\right|}_{p}^{\alpha }\\ & & -\frac{1}{24}\sum_{\overset{1\leq i,j,k,l\leq 4}{i\neq j\neq k\neq l}}{\left|X_{i}+Y_{j}-X_{k}-Y_{l}\right|}_{p}^{\alpha }\\ & & -\frac{1}{12}\sum_{\overset{1\leq i,j\leq 4}{i\neq j}}{\left|X_{i}+Y_{i}-X_{j}-Y_{j}\right|}_{p}^{\alpha }. \end{array}$$

Given *n* samples $\left(X_{1},Y_{1}\right),\cdots ,\left(X_{n},Y_{n}\right)$ from (X,Y), an estimator of $siCov^{\left(\alpha \right)}\left(X,Y\right)$ can be defined as

$$\begin{array}{c} {\hat{siCov^{\left(\alpha \right)}}}_{n}\left(X,Y\right)=\frac{1}{C_{n}^{4}}\sum_{1\leq i<j<k<l\leq n}k\left(\left(X_{i},Y_{i}\right),\left(X_{j},Y_{j}\right),\left(X_{k},Y_{k}\right),\left(X_{l},Y_{l}\right)\right). \end{array}$$

We directly obtain $E\left({\hat{siCov^{\left(\alpha \right)}}}_{n}\left(X,Y\right)\right)=siCov^{\left(\alpha \right)}\left(X,Y\right)$; that is, ${\hat{siCov^{\left(\alpha \right)}}}_{n}\left(X,Y\right)$ is an unbiased estimate of $siCov^{\left(\alpha \right)}\left(X,Y\right)$.

Using the general theory of *U*-statistics (see, e.g., Serfling [22] [p. 190, Theorem A]), we have the following result.

**Theorem** **6.** *Suppose that ${E|X|}_{p}^{\alpha }+E{\left|Y\right|}_{p}^{\alpha }<\infty$. Given n samples $(X_{1},Y_{1}),$$\cdots ,(X_{n},Y_{n})$ from (X,Y),*

$${\hat{siCov^{\left(\alpha \right)}}}_{n}\left(X,Y\right)\overset{a.s.}{⟶}siCov^{\left(\alpha \right)}\left(X,Y\right),\;as\;n\to \infty ,$$

*and*

$${\hat{siCor^{\left(\alpha \right)}}}_{n}\left(X,Y\right)\overset{a.s.}{⟶}siCor^{\left(\alpha \right)}\left(X,Y\right),\;as\;n\to \infty .$$

The following theorem gives the asymptotic distribution of the test statistic based on a known result on the convergence of *U*-statistics (Serfling [22] [Chapter 5.5.1, Theorem A]). We state the result without a proof.

**Theorem** **7.** *Suppose that $0<Var(k_{1}\left(X,Y\right))<\infty$. Given n samples $\left(X_{1},Y_{1}\right),\cdots ,\left(X_{n},Y_{n}\right)$ from (X,Y),*

$$n^{\frac{1}{2}}\left({\hat{siCov^{\left(\alpha \right)}}}_{n}\left(X,Y\right)-siCov^{\left(\alpha \right)}\left(X,Y\right)\right)\overset{D}{⟶}N\left(0,16Var\left(\left(k_{1}\left(X,Y\right)\right)\right)\right),$$

*as n→∞, where*

$$k_{1}\left(\left(x_{1},y_{1}\right)\right)=E_{2,3,4}k\left(\left(x_{1},y_{1}\right),\left(X_{2},Y_{2}\right),\left(X_{3},Y_{3}\right),\left(X_{4},Y_{4}\right)\right).$$

*Here, $E_{2,3,4}$ stands for taking expectation over $\left(X_{2},Y_{2}\right),\left(X_{3},Y_{3}\right),\left(X_{4},Y_{4}\right)$.*

If $0<Var(k_{1}\left(X,Y\right))<\infty$, then *X* and *Y* cannot be sub-independent; otherwise, $k_{1}\left(X,Y\right)=0$ almost surely. When *X* and *Y* are sub-independent, the asymptotic distribution of

$$n^{\frac{1}{2}}\left({\hat{siCov^{\left(\alpha \right)}}}_{n}\left(X,Y\right)-siCov^{\left(\alpha \right)}\left(X,Y\right)\right)$$

is no longer normal. The asymptotic distribution can be derived using results on first-order degenerate *U*-statistics.

**Theorem** **8.** *Suppose that X and Y are sub-independent with ${E|X|}_{p}^{\alpha }+E{\left|Y\right|}_{p}^{\alpha }<\infty$. Given n samples $\left(X_{1},Y_{1}\right),\cdots ,\left(X_{n},Y_{n}\right)$ from (X,Y),*

$$n^{\frac{1}{2}}{\hat{siCov^{\left(\alpha \right)}}}_{n}\left(X,Y\right)\overset{D}{⟶}6\sum_{i=1}^{\infty }\lambda_{i}\left(\chi_{i}^{2}-1\right),\;as\;n\to \infty ,$$

*where $\chi_{i}^{2}$’s are i.i.d. $\chi^{2}\left(1\right)$ variates and $\left\{\lambda_{i}\right\}$ are the eigenvalues of operator G, which is defined as $Gf\left(x_{1},y_{1}\right)=E\left(k_{12}\left(x_{1},y_{1}\right),\left(X_{2},Y_{2}\right)f\left(X_{2},Y_{2}\right)\right),$ where*

$$k_{12}\left(\left(x_{1},y_{1}\right),\left(x_{2},y_{2}\right)\right)=E_{3,4}k\left(\left(x_{1},y_{1}\right),\left(x_{2},y_{2}\right),\left(X_{3},Y_{3}\right),\left(X_{4},Y_{4}\right)\right).$$

*Here, $E_{3,4}$ stands for taking expectation over $\left(X_{3},Y_{3}\right),\left(X_{4},Y_{4}\right)$.*

**Proof.**  The asymptotic distribution of ${\hat{siCov^{\left(\alpha \right)}}}_{n}\left(X,Y\right)$ can be proved by modifying the proof of the theorem of Serfling [22] [p. 194]. □

### 5.1. A Simulation Study

We next examine the finite-sample performance of the estimator given above. We consider the two distributions in Section 4. For the normal case, let (X,Y) have the bivariate standard normal distribution $N_{2}\left(0,0,1,1,\rho \right)$ with ρ=0.5 and take α=1. For the Cauchy case, let $\left(X,Y\right)∼MC_{2}\left(0,I_{2}\right)$ and take α=0.5. The sample sizes are n=20,50,100,200,500,1000, and each setting is repeated 1000 times. The theoretical values of $siCov^{\left(\alpha \right)}\left(X,Y\right)$ and $siCor^{\left(\alpha \right)}\left(X,Y\right)$ are calculated from the formulas in Section 4.1 and Section 4.2.

Let ${\hat{siCov^{\left(\alpha \right)}}}_{n,b}\left(X,Y\right)$ denote the estimate from the *b*th replication, b=1,…,B, and let $siCov^{\left(\alpha \right)}\left(X,Y\right)$ denote the corresponding theoretical value. The Monte Carlo mean, bias, variance, and root mean squared error (RMSE) are defined, respectively, by

$$\begin{array}{cc} {\bar{siCov}}_{n}^{\left(\alpha \right)}\left(X,Y\right) & =\frac{1}{B}\sum_{b=1}^{B}{\hat{siCov^{\left(\alpha \right)}}}_{n,b}\left(X,Y\right);\\ \mathrm{Bias} & ={\bar{siCov}}_{n}^{\left(\alpha \right)}\left(X,Y\right)-siCov^{\left(\alpha \right)}\left(X,Y\right);\\ \mathrm{Variance} & =\frac{1}{B-1}\sum_{b=1}^{B}{\left[{\hat{siCov^{\left(\alpha \right)}}}_{n,b}\left(X,Y\right)-{\bar{siCov}}_{n}^{\left(\alpha \right)}\left(X,Y\right)\right]}^{2};\\ \mathrm{RMSE} & ={\left\{\frac{1}{B}\sum_{b=1}^{B}{\left[{\hat{siCov^{\left(\alpha \right)}}}_{n,b}\left(X,Y\right)-siCov^{\left(\alpha \right)}\left(X,Y\right)\right]}^{2}\right\}}^{1/2}. \end{array}$$

**Figure 1:**
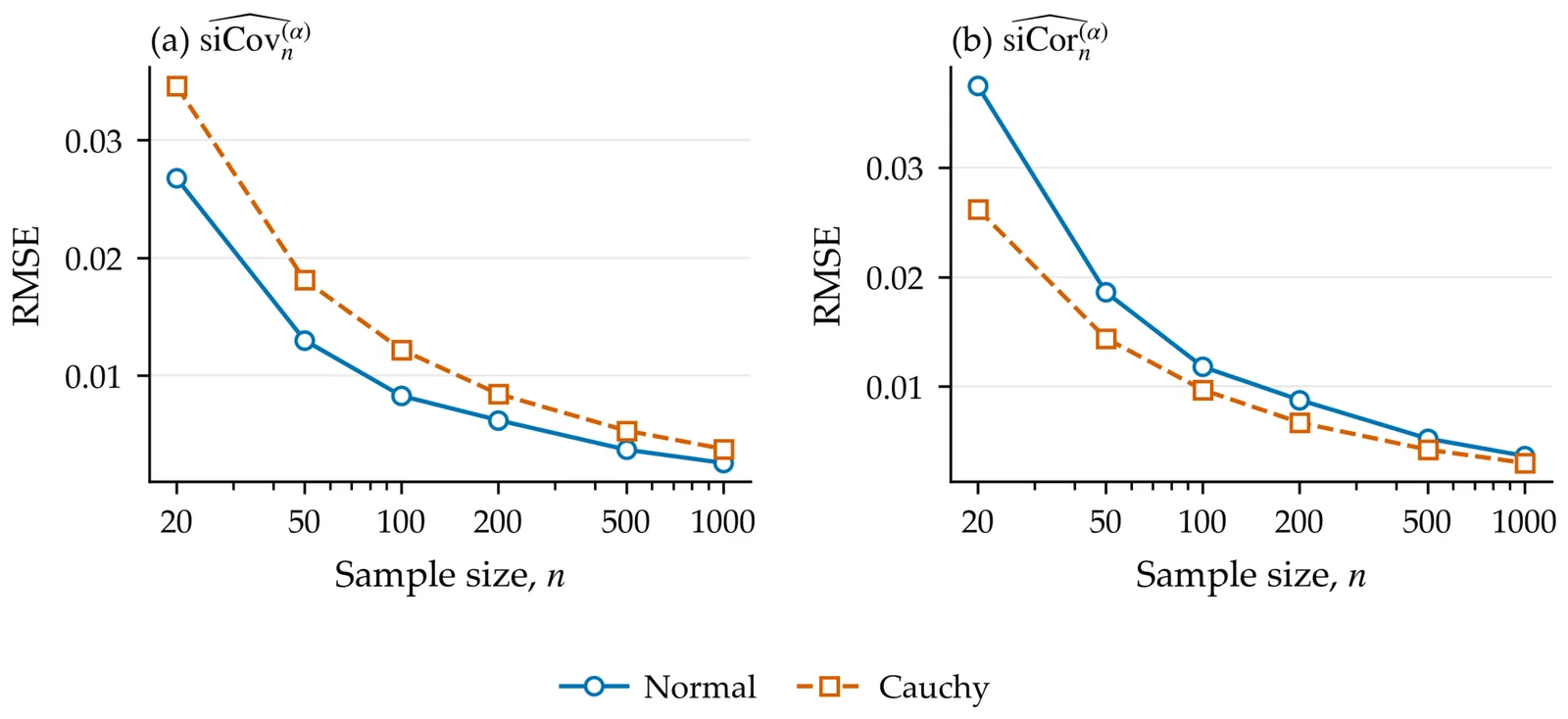
RMSE of the estimators under the bivariate normal and Cauchy distributions. (**a**) ${\hat{siCov^{\left(\alpha \right)}}}_{n}\left(X,Y\right)$; (**b**) ${\hat{siCor^{\left(\alpha \right)}}}_{n}\left(X,Y\right)$.

For each setting, we calculate these four quantities for ${\hat{siCov^{\left(\alpha \right)}}}_{n}\left(X,Y\right)$. The results are reported in Table 1. The same quantities are also calculated for ${\hat{siCor^{\left(\alpha \right)}}}_{n}\left(X,Y\right)$, and the corresponding RMSEs are shown in Figure 1.

For the normal distribution, the variance and RMSE of both estimators decrease as the sample size increases, and the mean estimates remain close to the theoretical values. The variance and RMSE also decrease for the Cauchy distribution.

### 5.2. Comparison with Other Dependence Measures

We further compare Pearson correlation, siCor and squared distance correlation. In addition to independent standard normal variables, we consider a sub-independent but dependent example. Let X,Y∈{0,1,2} have joint probability matrix

(12) $$P_{\delta }=\left(\begin{array}{ccc} \frac{1}{9} & \frac{1}{9}-\delta & \frac{1}{9}+\delta \\ \frac{1}{9}+\delta & \frac{1}{9} & \frac{1}{9}-\delta \\ \frac{1}{9}-\delta & \frac{1}{9}+\delta & \frac{1}{9} \end{array}\right),\;\delta =\frac{1}{12}.$$

**Figure 2:**
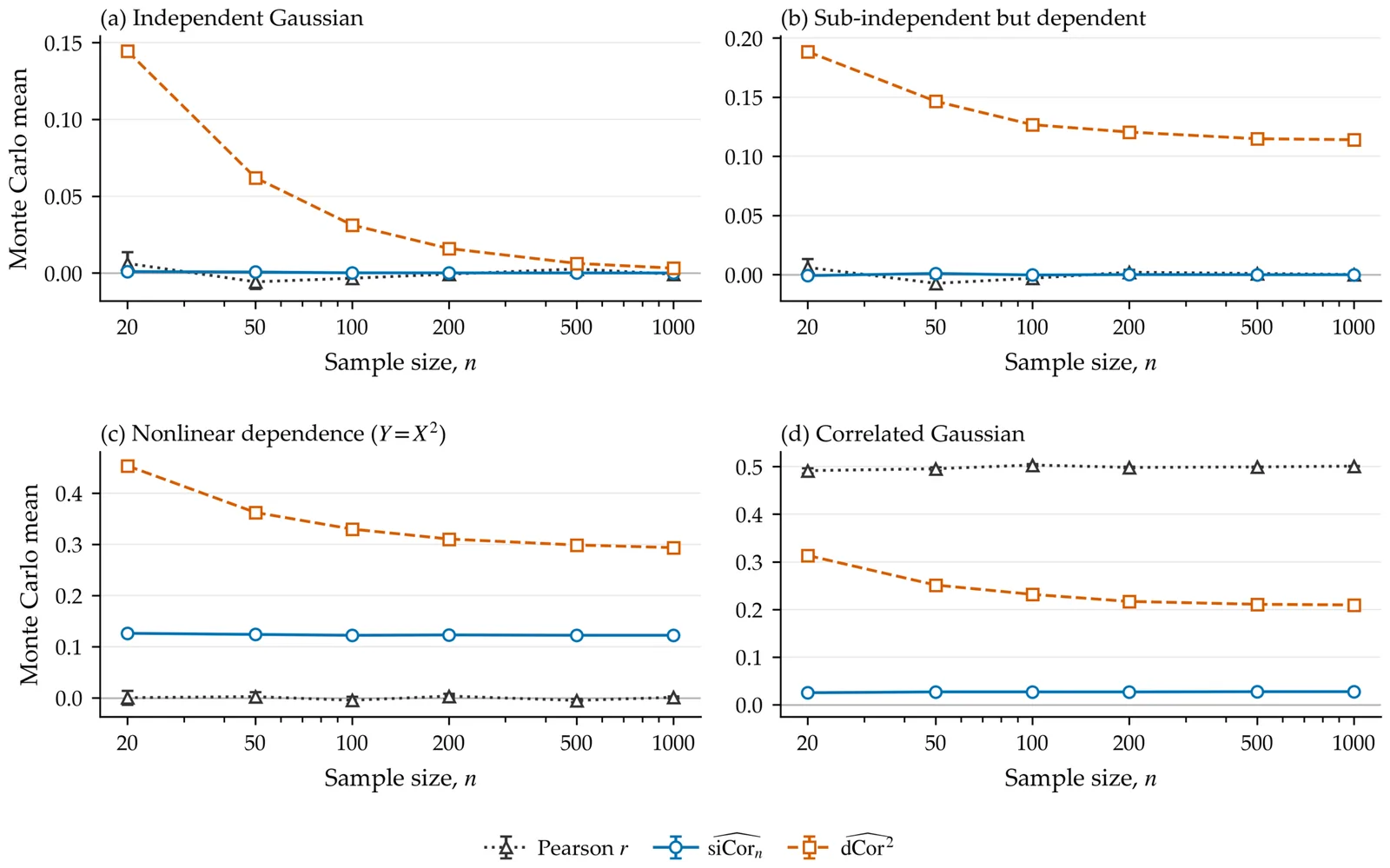
Comparison of Pearson correlation, siCor, and squared distance correlation as the sample size changes. (**a**) Independent normal variables; (**b**) sub-independent but dependent variables; (**c**) nonlinear dependence with zero Pearson correlation; (**d**) correlated normal variables.

The marginal distributions are uniform, and the distribution of X+Y is the convolution of the marginal distributions, while *X* and *Y* are dependent. We also use the nonlinear model X∼N(0,1), $Y=X^{2}$, and the bivariate standard normal distribution with ρ=0.5. For siCor, we take α=1. The sample sizes are n=20,50,100,200,500,1000, and each setting is repeated 1000 times. The results are reported in Table 2 and Figure 2.

For the independent normal variables, the three sample measures approach zero as the sample size increases. In the sub-independent but dependent case, Pearson correlation and siCor remain close to zero, while squared distance correlation remains positive. For $Y=X^{2}$, Pearson correlation remains close to zero, whereas siCor and squared distance correlation are positive. For the correlated normal variables, all three measures are positive. These results are consistent with the relationships discussed in Section 3.

## 6. Applications

Many well-known results in probability and statistics are often based on the distribution of the sums of independent (and often identically distributed) random variables rather than the joint distribution of the summands. In other words, it is the convolution of the marginal distributions, as in the case of independence, that is needed rather than the joint distribution of the summands. Therefore, the full strength of independence of the summands will not be required. The concept of sub-independence can replace that of independence in most of the theorems in these situations, which deal with the distribution of the sum of random variables. For example, in Khintchine’s law of large numbers and Lindeberg–Lévy’s central limit theorem as well as other important theorems in probability and statistics (cf. summability of random variables, compound of Poisson processes, renewal theorems, and so on), we have only used the characteristic functions of the sum of random variables $X_{i}$’s. More precisely, the proof does not employ the full power of independence of $X_{i}$’s; it only uses the fact that $\phi_{X_{1}+\cdots +X_{n}}\left(t\right)=\prod_{i=1}^{n}\phi_{X_{i}}\left(t\right)$ for all t∈R. Therefore, the assumption of independent and identical distribution throughout this paper can be replaced with that of sub-independent and identical distribution whenever appropriate. For more details, see Hamedani and Maadooliat [23]. We mention below a few results in which the assumption of independence is replaced by that of sub-independence.

### 6.1. Concentration Inequalities in Machine Learning

Concentration inequalities give probability bounds for a random variable to be concentrated around its mean, or for it to deviate from its mean or some other value. We present several concentration inequalities used in machine learning.

We start this subsection with the sub-independent version of Hoeffding’s inequality (Hoeffding [24] [Theorem 2]; see also Theorem D.2 in Mohri et al. [25]).

**Theorem** **9** (Hoeffding’s inequality)**.** *Let $X_{1},\cdots ,X_{n}$ be sub-independent random variables with $X_{i}$ taking values in $\left[a_{i},b_{i}\right]$(i=1,2,⋯,n). Then, for any ε>0, the following inequalities hold for $S_{n}=\sum_{i=1}^{n}X_{i}$:*

$$P\left(S_{n}-ES_{n}\geq \varepsilon \right)\leq e^{-2\varepsilon^{2}/\sum_{i=1}^{n}{\left(b_{i}-a_{i}\right)}^{2}},$$

*and*

$$P\left(S_{n}-ES_{n}\leq -\varepsilon \right)\leq e^{-2\varepsilon^{2}/\sum_{i=1}^{n}{\left(b_{i}-a_{i}\right)}^{2}}.$$

**Proof.**  The boundedness of $X_{i}$ on the finite interval $\left[a_{i},b_{i}\right]$ ensures integrability, so $Ee^{tX_{i}}$ is well-defined and finite for every *i*. We only note that the sub-independence of $X_{1},\cdots ,X_{n}$ implies that $\sum_{i=1}^{n}X_{i}\overset{st}{=}\sum_{i=1}^{n}X_{i}^{*}$, where $\overset{st}{=}$ denotes the stochastic equality. Here, $X_{1}^{*},X_{2}^{*},\cdots ,X_{n}^{*}$ are i.i.d. and $X_{i}^{*}\overset{st}{=}X_{i}$. Hence, for t>0, $Ee^{tS_{n}}=\prod_{i=1}^{n}Ee^{tX_{i}}$. The rest follows from the proof given in Mohri et al. [25]. □

The following theorem presents a finer upper bound than Hoeffding’s inequality expressed in terms of the binary relative entropy.

**Theorem** **10** (Sanov’s theorem)**.** *Let $X_{1},\cdots ,X_{n}$ be sub-independent random variables drawn according to some distribution F with mean p and support included in [0,1]. Then, for any q∈[0,1], the following inequality holds for $\hat{p}=1/n\sum_{i=1}^{n}X_{i}$:*

$$P\left(\hat{p}\geq q\right)\leq e^{-nD(q||p)},$$

*where $D\left(q||p\right)=q\log\frac{q}{p}+\left(1-q\right)\log\frac{1-q}{1-p}$ is the binary relative entropy of p and q.*

Sanov’s theorem, based on the concept of sub-independence, can be used to prove the following multiplicative Chernoff bounds that strengthen the characterizations given in Mohri et al. [25] [Theorem D.4] under the assumption of independence.

**Theorem** **11** (Multiplicative Chernoff bounds)**.** *Let $X_{1},\cdots ,X_{n}$ be sub-independent random variables drawn according to some distribution F with mean p and support included in [0,1]. Then, for any γ∈[0,1/p−1], the following inequalities hold for $\hat{p}=1/n\sum_{i=1}^{n}X_{i}$:*

$$P\left(\hat{p}\geq \left(1+\gamma \right)p\right)\leq e^{-\frac{np\gamma^{2}}{3}},$$

*and*

$$P\left(\hat{p}\leq \left(1-\gamma \right)p\right)\leq e^{-\frac{np\gamma^{2}}{2}}.$$

### 6.2. The Sub-Independent Version of Actuarial Models

In this section, we present some important models and settings in actuarial science based on the concept of sub-independence, where it has traditionally been assumed that independence holds between certain risks and that the number of terms is independent of the summands (see, e.g., Kaas et al. [26]).

#### 6.2.1. The Individual Risk Model Under Sub-Independence

We consider a portfolio of *n* sub-independent insurance policies (or risks) $X_{i}$’s. The aggregate claim amount for the portfolio is $S=X_{1}+\cdots +X_{n}$. When the number *n* of policies is large, according to the sub-independence version of the central limit theorem, *S* can approximately follow a normal distribution

$$S∼N\left(\sum_{i=1}^{n}E\left(X_{i}\right),\sum_{i=1}^{n}Var\left(X_{i}\right)\right).$$

This approximation greatly improves computational efficiency, especially for the risk assessment of large-scale policy portfolios. For small-scale combinations, the distribution of *S* can be accurately solved through convolution operations:

$$F_{S}\left(s\right)=F_{X_{1}}*F_{X_{1}}*\cdots *F_{X_{n}}\left(s\right).$$

or using the moment generating function (MGF): $M_{S}\left(t\right)=\prod_{i=1}^{n}M_{X_{i}}\left(t\right);$ or using the characteristic function (CF): $\psi_{S}\left(t\right)=\prod_{i=1}^{n}\psi_{X_{i}}\left(t\right).$

We now present several examples of distributions possessing the reproductive property (i.e., closure under convolution) under sub-independence.

**Example** **1.**

*(i)*  *If $X_{1},\cdots ,X_{n}$ are sub-independent and $X_{i}∼Bin\left(n_{i},p\right),i=1,2,\cdots ,n$, then $\sum_{i=1}^{n}X_{i}∼Bin\left(\sum_{i=1}^{n}n_{i},p\right).$*
*(ii)*  *If $X_{1},\cdots ,X_{n}$ are sub-independent and $X_{i}∼Poi\left(\lambda_{i}\right),i=1,2,\cdots ,n$, then $\sum_{i=1}^{n}X_{i}∼Poi\left(\sum_{i=1}^{n}\lambda_{i}\right).$*
*(iii)*  *If $X_{1},\cdots ,X_{n}$ are sub-independent and $X_{i}∼NB\left(r_{i},p\right),i=1,2,\cdots ,n$, then $\sum_{i=1}^{n}X_{i}∼NB\left(\sum_{i=1}^{n}r_{i},p\right).$*
*(iv)*  *If $X_{1},\cdots ,X_{n}$ are sub-independent and $X_{i}∼N\left(\mu_{i},\sigma_{i}^{2}\right),i=1,2,\cdots ,n$, then $\sum_{i=1}^{n}X_{i}∼N\left(\sum_{i=1}^{n}\mu_{i},\sum_{i=1}^{n}\sigma_{i}^{2}\right)$.*

*There are similar conclusions about gamma, chi-square and Cauchy distribution as well.*

#### 6.2.2. The Collective Risk Model Under Sub-Independence

The total amount paid (or aggregate losses) incurred by an insurer is expressed as $S_{N}=X_{1}+\cdots +X_{N}$, where $X_{1},X_{2},\cdots$ are sub-independent identically distributed random variables representing the claim payment amounts and *N* is the (random) number of claims incurred with the probability mass function $P\left(N=n\right)=p_{n},n=0,1,\cdots$. Throughout, we set $S_{0}=0$, $M_{X}\left(t\right)=E\left(e^{tX}\right),P_{X}\left(z\right)=E\left(z^{X}\right)$. We assume that $E\left(e^{itS_{n}}|N\right)=E\left(e^{itS_{n}}\right)$ for all real *t*. Then, the distribution of *S* is given as $F_{S}\left(s\right)=\sum_{n=0}^{\infty }F_{X}^{*n}\left(s\right)p_{n},$ the probability generating function of *S* is given as $P_{S}\left(z\right)=P_{N}{\left(P_{X}\left(z\right)\right)}^{n}),$ the moment generating function of *S* is given as $M_{S}\left(t\right)=P_{N}\left(M_{X}\left(t\right)\right),$ and the characteristic function of *S* is given as $\psi_{S}\left(t\right)=P_{N}\left(\psi_{X}\left(t\right)\right).$ In particular,

$$ES_{N}=E\left(N\right)E\left(X\right),\;Var\left(S_{N}\right)=E\left(N\right)Var\left(X\right)+{\left(EX\right)}^{2}Var\left(N\right).$$

**Remark** **3.** *In particular, if N∼Poisson(λ), i.e., $P\left(N=n\right)=\frac{e^{-\lambda }\lambda^{n}}{n!},\;n=0,1,2,\cdots$. The aggregate claim amount $S_{N}$ follows a compound Poisson distribution with rate λ and severity distribution $F_{X}$: $S_{N}∼$ Compound Poisson $\left(\lambda ,F_{X}\right)$. The characteristic function of $S_{N}$ is given as*

$$\psi_{S_{N}}\left(t\right)=\exp\left(\lambda \left(\psi_{X}\left(t\right)-1\right)\right)).$$

*From this, we can prove that the sum of n sub-independent compound Poisson distributions is still a compound Poisson distribution.*

### 6.3. The Sub-Independent Version of Renewal Theorems

Let $X,X_{1},X_{2},\cdots$ be a sequence of non-negative, independent identically distributed (i.i.d.) random variables (r.v.’s) with distribution function (d.f.) F(x)=P(X≤x) and expectation μ. The sequence $\left\{S_{n},n=0,1,2,...\right\}$ defined by $S_{0}=0$ and $S_{n}=X_{1}+\cdots +X_{n},n=1,2,\ldots$, is called a renewal sequence or a renewal process. The renewal counting process {N(t),t≥0} associated with the renewal sequence $\left\{S_{n},n\geq 0\right\}$ is defined by $N\left(t\right)=\sup\left\{n\geq 0:S_{n}\leq t\right\}$. Most of the important classical results in renewal theory based on the concept of independence can be stated in terms of sub-independent rv’s. We only list some of them.

The following result for i.i.d. random variables is well-known; see, e.g., Mitov and Omey [27].

**Theorem** **12.** *Let $X_{1},X_{2},\cdots$ be a sequence of non-negative, sub-independent identically distributed random variables with a proper d.f. F; then,*

*(i) with probability 1,*

$$\begin{array}{c} \lim_{t\to \infty }\frac{N(t)}{t}=\left\{\begin{array}{cc} \frac{1}{\mu },\; & \mathrm{if}\;\;\mu <\infty ,\\ 0,\; & \mathrm{if}\;\;\mu =\infty ; \end{array}\right. \end{array}$$

*(ii)*

$$P\left(N\left(t\right)=n\right)=F^{*n}\left(t\right)-F^{*n+1}\left(t\right),n=1,2,\cdots ,$$

*where $F^{*n}$ is the n-fold convolution of F with itself.*

The following theorem gives the results of the process N(t) under the assumption of sub-independence.

**Theorem** **13.** *Let $X_{1},X_{2},\cdots$ be a sequence of non-negative, sub-independent identically distributed random variables with a proper d.f. F satisfying F(0)<1 and F(∞)=1; then,*

(13) $$U\left(t\right):=E\left(N\left(t\right)\right)=\sum_{k=1}^{\infty }F^{*k}\left(t\right).$$

U(t)=F(t)+(U∗F)(t),

*and*

$$\begin{array}{c} \lim_{t\to \infty }\frac{U(t)}{t}=\left\{\begin{array}{cc} \frac{1}{\mu },\; & \mathrm{if}\;\;\mu <\infty ,\\ 0,\; & \mathrm{if}\;\;\mu =\infty , \end{array}\right. \end{array}$$

*where $F^{*n}$ is the n-fold convolution of F with itself. If, in addition, $0<\sigma^{2}=Var\left(X\right)<\infty$, then*

$$\frac{N(t)-t/\mu }{\sigma \sqrt{\mu^{3}t}}\overset{D}{⟶}N\left(0,1\right),\;as\;t\to \infty .$$

### 6.4. An Application to International Financial Data

We finally give a real-data application of the proposed measure. We consider local-currency stock returns and foreign exchange returns because their sum has a direct interpretation in a common currency. Let $P_{j,t}$ be the closing level of the stock index in country *j*, and let $E_{j,t}$ be the exchange rate expressed as US dollars per unit of local currency. Define

$$\begin{array}{ccc} R_{j,t}^{S} & = & 100\left(\log P_{j,t}-\log P_{j,t-1}\right),\\ R_{j,t}^{F} & = & 100\left(\log E_{j,t}-\log E_{j,t-1}\right). \end{array}$$

Since the US dollar value of the stock index is $P_{j,t}E_{j,t}$, the corresponding log return is

(14) $$\begin{array}{ccc} R_{j,t}^{USD} & = & 100\left[\log\left(P_{j,t}E_{j,t}\right)-\log\left(P_{j,t-1}E_{j,t-1}\right)\right]\\ & = & R_{j,t}^{S}+R_{j,t}^{F}. \end{array}$$

This decomposition of stock returns in a common currency is also used in Mink [28].

We use the FTSE 100 index for the United Kingdom, the Nikkei 225 index for Japan, the S&P/ASX 200 index for Australia, and the S&P/TSX Composite index for Canada. The corresponding exchange rates are GBP/USD, JPY/USD, AUD/USD, and CAD/USD. Daily closing index levels and exchange rates are obtained from Yahoo Finance. For each month, we use the last common trading date of the eight series and calculate monthly log returns. The sample period is from January 2016 to December 2025 and contains 120 monthly observations.

Let

$$\begin{array}{ccc} X_{t} & = & {\left(R_{UK,t}^{S},R_{JP,t}^{S},R_{AU,t}^{S},R_{CA,t}^{S}\right)}^{\prime },\\ Y_{t} & = & {\left(R_{GBP,t}^{F},R_{JPY,t}^{F},R_{AUD,t}^{F},R_{CAD,t}^{F}\right)}^{\prime }. \end{array}$$

**Figure 3:**
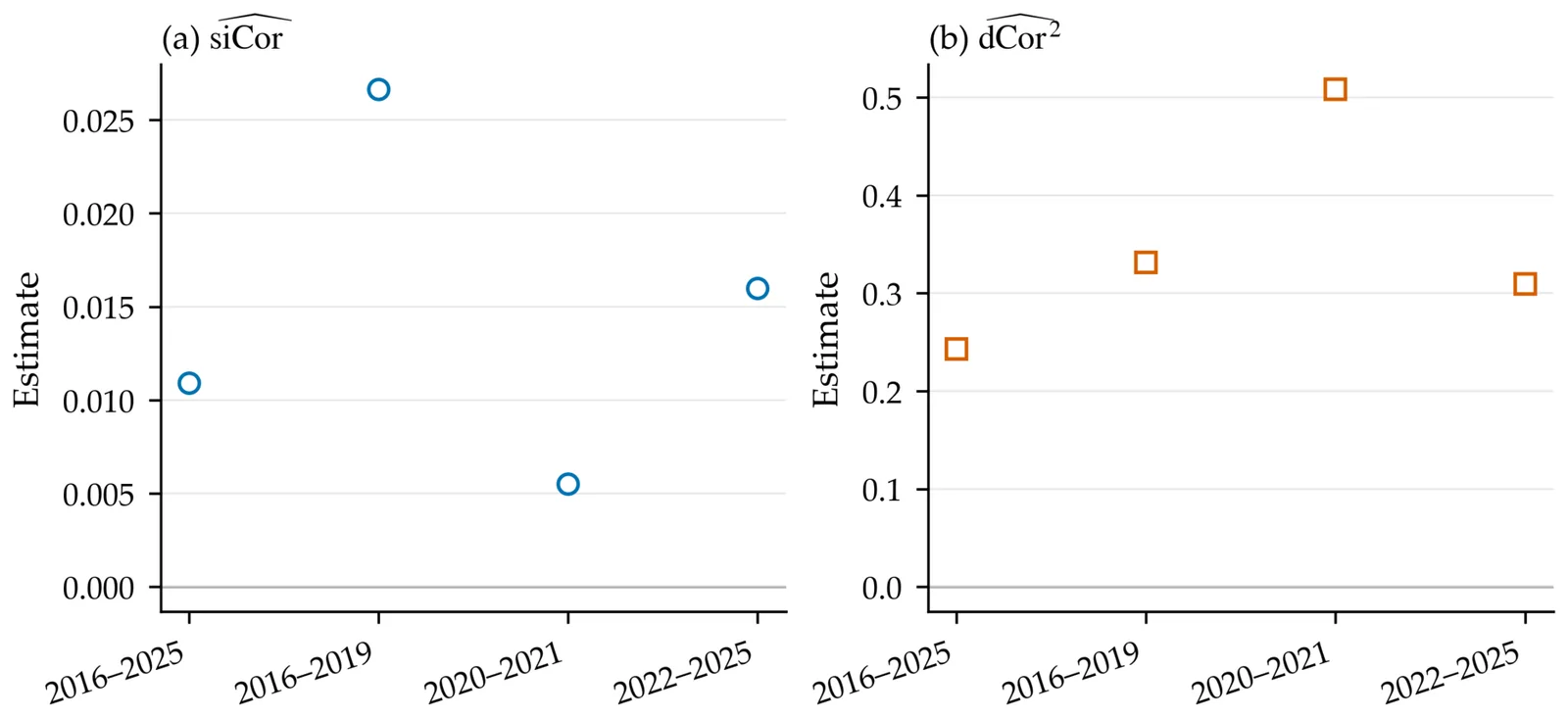
Sub-independent correlation and squared distance correlation for the full sample and three subperiods of the international financial data. (**a**) Sub-independent correlation ${\hat{siCor}}_{n}$; (**b**) squared distance correlation ${\hat{\mathrm{dCor}}}^{\;2}$.

Then, $X_{t}+Y_{t}$ is the four-dimensional vector of US dollar stock index log returns. We calculate ${\hat{siCov}}_{n}\left(X,Y\right)$ and ${\hat{siCor}}_{n}\left(X,Y\right)$ with α=1 using the estimator in Section 5. For comparison, we also calculate the squared sample distance correlation. The results for 2016–2019, 2020–2021, and 2022–2025 are also reported. Table 3 gives the results. Figure 3 displays the corresponding values of ${\hat{siCor}}_{n}$ and ${\hat{\mathrm{dCor}}}^{\;2}$.

For the full sample, ${\hat{siCor}}_{n}\left(X,Y\right)=0.010913$, while ${\hat{\mathrm{dCor}}}^{\;2}\left(X,Y\right)=0.243059$. The same difference appears in the three subperiods. In 2020–2021, for example, ${\hat{siCor}}_{n}\left(X,Y\right)=0.005508$ and ${\hat{\mathrm{dCor}}}^{\;2}\left(X,Y\right)=0.508325$. Thus, the squared distance correlation is considerably larger than the sub-independent correlation. Since $X_{t}+Y_{t}$ is the US dollar stock return vector, the proposed measure describes the dependence associated with the distribution of this sum.

## 7. Conclusions and Prospects 

This paper introduces a new index of dependence for testing sub-independence. The simulation results for the bivariate normal and Cauchy distributions show that the variance and RMSE of the estimator decrease as the sample size increases. The comparative simulations also show different patterns for Pearson correlation, siCor, and squared distance correlation under the four dependence structures considered. The financial data application gives a small sub-independent correlation between the stock and foreign exchange return vectors, while the squared distance correlation is larger. The concept of sub-independence lies in between independence and uncorrelatedness and is much weaker than that of independence, and is shown to be sufficient to yield the conclusions of theorems and results in these situations that deal with the distribution of the sum of random variables. Hence, it provides a more flexible theoretical basis for handling nonlinear and non-normally distributed data. Sub-independence can be applied in modern statistical models for complex scenarios where traditional independence assumptions are too strong and only uncorrelated assumptions are insufficient to characterize relationships. It can be expected to demonstrate unique value in the following areas:

**Financial time-series modeling**: In asset return modeling, the independence of sequences often does not hold (such as volatility clustering effects), but assuming complete correlation directly would make the model too complex. Sub-independence can be used to describe weak dependencies between high-order moments (such as volatility), capturing nonlinear dynamic features while maintaining model simplicity. It is commonly used in residual diagnosis and extensions of GARCH family models.

**Survival analysis and reliability engineering**: In multivariate survival data, failure events between individuals may not be completely independent (such as familial genetic influences), but there may not necessarily be strong associations. Sub-independence is used to construct a semi-parametric copula model, allowing for the flexible setting of marginal distributions while connecting variables with weaker dependency structures to enhance model robustness.

**Dimensionality reduction and variable selection:** In gene expression data analysis, there are complex regulatory networks among thousands of genes. Sub-independence can be used as a screening criterion to identify gene modules that are not completely independent but have potential functional associations, assisting in the construction of more reasonable biological network models.

**Bayesian networks and causal inference**: When the data is insufficient to support strong independence testing, secondary independence can be used as an intermediate hypothesis to simplify the learning process of graph model structure. It helps to avoid structural misjudgment caused by excessive reliance on chi-square tests or information criteria, especially suitable for small-sample or sparse-data scenarios.

**Feature engineering in machine learning**: In ensemble learning and random forests, the selection of feature subsets often relies on the assumption of independence. Introducing sub-independence measures can more finely control the redundancy between features and improve the model’s generalization ability while ensuring diversity.

## Figures and Tables

**Table 1:** Finite-sample performance of ${\hat{siCov^{\left(\alpha \right)}}}_{n}\left(X,Y\right)$ and ${\hat{siCor^{\left(\alpha \right)}}}_{n}\left(X,Y\right)$ under the bivariate normal and Cauchy distributions.

Distribution	α	*n*	True Value	Mean	Bias	Variance	RMSE
*Panel A: ${\hat{siCov^{\left(\alpha \right)}}}_{n}\left(X,Y\right)$*							
Normal	1.0	20	0.018069	0.018340	0.000271	0.000718	0.026776
Normal	1.0	50	0.018069	0.017394	−0.000675	0.000168	0.012991
Normal	1.0	100	0.018069	0.017975	−0.000094	0.000068	0.008270
Normal	1.0	200	0.018069	0.018402	0.000333	0.000038	0.006199
Normal	1.0	500	0.018069	0.017893	−0.000176	0.000014	0.003691
Normal	1.0	1000	0.018069	0.017989	−0.000080	0.000007	0.002568
Cauchy	0.5	20	0.019411	0.019910	0.000499	0.001198	0.034602
Cauchy	0.5	50	0.019411	0.018636	−0.000775	0.000328	0.018118
Cauchy	0.5	100	0.019411	0.019962	0.000551	0.000149	0.012200
Cauchy	0.5	200	0.019411	0.019385	−0.000026	0.000071	0.008430
Cauchy	0.5	500	0.019411	0.019713	0.000303	0.000028	0.005311
Cauchy	0.5	1000	0.019411	0.019486	0.000075	0.000014	0.003758
*Panel B: ${\hat{siCor^{\left(\alpha \right)}}}_{n}\left(X,Y\right)$*							
Normal	1.0	20	0.027336	0.026090	−0.001246	0.001402	0.037444
Normal	1.0	50	0.027336	0.025832	−0.001504	0.000345	0.018616
Normal	1.0	100	0.027336	0.026896	−0.000440	0.000139	0.011804
Normal	1.0	200	0.027336	0.027664	0.000328	0.000077	0.008779
Normal	1.0	500	0.027336	0.027040	−0.000297	0.000028	0.005255
Normal	1.0	1000	0.027336	0.027199	−0.000137	0.000013	0.003669
Cauchy	0.5	20	0.016568	0.015534	−0.001034	0.000686	0.026201
Cauchy	0.5	50	0.016568	0.015450	−0.001118	0.000207	0.014408
Cauchy	0.5	100	0.016568	0.016786	0.000218	0.000094	0.009706
Cauchy	0.5	200	0.016568	0.016373	−0.000195	0.000045	0.006715
Cauchy	0.5	500	0.016568	0.016756	0.000188	0.000018	0.004240
Cauchy	0.5	1000	0.016568	0.016620	0.000053	0.000009	0.003028

*Note:* The normal distribution has ρ=0.5 and the Cauchy distribution is $MC_{2}\left(0,I_{2}\right)$. Each setting is based on 1000 replications. Bias is the Monte Carlo mean minus the theoretical value.

**Table 2:** Monte Carlo comparison of Pearson correlation, siCor, and squared distance correlation under different dependence structures.

*n*	Pearson *r*	${\hat{{\mathrm{siCor}}^{\left(1\right)}}}_{n}$	${\hat{\mathrm{dCor}}}^{\;2}$
*Panel A: Independent normal*			
20	0.006259 (0.231886)	0.000919 (0.028377)	0.144493 (0.063283)
50	−0.005749 (0.144442)	0.000619 (0.010775)	0.062096 (0.027224)
100	−0.003399 (0.102665)	0.000115 (0.004913)	0.031256 (0.013874)
200	−0.000736 (0.072506)	0.000048 (0.002598)	0.015976 (0.006888)
500	0.002592 (0.043103)	0.000014 (0.001017)	0.006254 (0.002651)
1000	−0.000803 (0.031834)	0.000003 (0.000493)	0.003142 (0.001371)
*Panel B: Sub-independent but dependent*			
20	0.006168 (0.226716)	−0.000780 (0.051458)	0.188442 (0.097822)
50	−0.007299 (0.147392)	0.001046 (0.020854)	0.146538 (0.056409)
100	−0.002998 (0.101327)	−0.000258 (0.008740)	0.126735 (0.032842)
200	0.002084 (0.073181)	0.000095 (0.004805)	0.120509 (0.023071)
500	0.000978 (0.044590)	−0.000028 (0.001686)	0.114879 (0.013962)
1000	0.000161 (0.031033)	−0.000019 (0.000854)	0.114023 (0.009741)
*Panel C: Nonlinear uncorrelated*			
20	0.000313 (0.435543)	0.126021 (0.069834)	0.453301 (0.116627)
50	0.002372 (0.290577)	0.123885 (0.036912)	0.362211 (0.061639)
100	−0.004701 (0.221510)	0.122140 (0.024536)	0.329643 (0.036409)
200	0.003357 (0.161913)	0.122921 (0.016610)	0.310253 (0.021675)
500	−0.004983 (0.101497)	0.122194 (0.010334)	0.298565 (0.011522)
1000	0.001001 (0.068361)	0.122204 (0.007349)	0.293460 (0.007276)
*Panel D: Correlated normal*			
20	0.491466 (0.174685)	0.025194 (0.034765)	0.313449 (0.130450)
50	0.495504 (0.110611)	0.026948 (0.019251)	0.251277 (0.094626)
100	0.503287 (0.074330)	0.026910 (0.012582)	0.231697 (0.066691)
200	0.498283 (0.054682)	0.026857 (0.008268)	0.216858 (0.048361)
500	0.499309 (0.033048)	0.027349 (0.005189)	0.210915 (0.030249)
1000	0.500965 (0.023336)	0.027450 (0.003665)	0.209489 (0.021350)

*Note:* Entries are Monte Carlo means, with standard deviations in parentheses. Each setting is based on 1000 replications.

**Table 3:** Sub-independent measures and distance correlation for international stock and foreign exchange returns.

Period	*n*	${\hat{\mathrm{siCov}}}_{n}$	${\hat{\mathrm{siCor}}}_{n}$	${\hat{\mathrm{dCor}}}^{\;2}$
2016–2025	120	0.046559	0.010913	0.243059
2016–2019	48	0.104930	0.026647	0.331382
2020–2021	24	0.023949	0.005508	0.508325
2022–2025	48	0.071694	0.016003	0.309442

*Note: X* denotes the vector of local-currency stock index log returns and *Y* denotes the vector of foreign exchange log returns. The estimates of siCov and siCor are calculated with α=1.

## Data Availability

The data used in the empirical application are publicly available from Yahoo Finance at https://finance.yahoo.com/ (accessed on 29 September 2026).
